# Heavy Metals and the Occurrence of Ulcerative Dermal Necrosis (UDN) in Sea Trout from the RIVER REGA, Poland—Consumer Health Assessment

**DOI:** 10.3390/ijerph19042296

**Published:** 2022-02-17

**Authors:** Monika Rajkowska-Myśliwiec, Mikołaj Protasowicki, Adam Tański, Sandra Watrak

**Affiliations:** 1 Department of Toxicology, Dairy Technology and Food Storage, Faculty of Food Science and Fisheries, West Pomeranian University of Technology in Szczecin, 71-459 Szczecin, Poland; mikolaj.protasowicki@zut.edu.pl (M.P.); s.watrak@gmail.com (S.W.); 2 Department of Hydrobiology, Ichthyology and Reproductive Biotechnology, Faculty of Food Science and Fisheries, West Pomeranian University of Technology in Szczecin, 71-550 Szczecin, Poland; adam.tanski@zut.edu.pl

**Keywords:** heavy metals, see trout, UDN disease, dietary exposure, risk assessment, THQ, EDI, BMDL, TWI

## Abstract

Various factors, including heavy metals, can make fish more susceptible to disease. This study investigated, inter alia, the relationship between Pb, Cd, and Hg contamination of the organs and muscles of sea trout from the river Rega (Poland) and the occurrence of UDN (ulcerative skin necrosis) symptoms. Moreover, the dietary risk of Pb, Cd, and Hg uptake from the muscles of healthy sea trout was assessed based on estimated daily intake (EDI), target hazard quotient (THQ), benchmark dose lower confidence limits (BMDL_01_ and BMDL_10_), and tolerable weekly intake (TWI). The metal concentrations varied, depending on the fish organs and the fish condition. A significantly higher amount of Pb was found in the gonads, gills, and muscles, Cd in the kidneys, and Hg in the muscles of sea trout with UDN symptoms. The lowest concentrations of Pb and Cd were detected in the muscles, and of Hg in the gonads. Dietary exposure to Pb, Cd, and Hg accounted for: 0.4% of BMDL_01_ and 1.0% of BMDL_10_, 0.56% of TWI, and 16.5% of TWI, respectively. THQs for each metal and the HI value for combined metals were below 1. The weakening of fish due to UDN-like disease probably increased the accumulation of Pb in the gonads, gills, and muscles, and of Cd in the kidneys, of the sea trout. As regards the maximum levels (MRLs), the muscles of healthy sea trout are safe for consumption. The risk assessment suggests no concern for the health of consumers.

## 1. Introduction

The contamination of water and sediment with high concentrations of heavy metals poses a serious threat due to their toxicity, long persistence, bioaccumulation, and biomagnification in the food chain. [1]. Metals induce early responses in fish, as evidenced by alterations at both structural and functional levels of different organs, including enzymatic and genetic effects, which affect the innate immune system of exposed fish and/or increase susceptibility to multiple types of disease [1,2]. The development of oxidative stress is the fundamental molecular mechanism of metal toxicity. This stress weakens the immune system, causes tissue and organ damage and growth defects, and reduces reproductive ability [3]. The sea trout (*Salmo trutta* m. *trutta* L.), as a predatory fish, is the final link in the food chain and can play an important role as a bio-indicator in monitoring heavy metal pollution. It is an anadromous species, that is, it migrates to saltwater environments to feed and returns to freshwater streams to spawn [4]. The nominal catches of sea trout in the Baltic Sea, including rivers, totalled 312 tons in 2018 (Poland 237 t). Sea trout are mainly caught in coastal and river areas and only to some extent in offshore areas [5]. In Poland, sea trout are caught in 14 rivers, among which the river Rega ranks third for fishing opportunities [6]. For several years, ulcerative dermal necrosis (UDN) has been observed in sea trout. A significant amount of information about UDN has been published, but its exact etiology is still unknown [1,7]. The onset of symptoms only occurs after fish migrate into freshwater. According to a review by Roberts [8], UDN was first reported at the end of the 19th century in Great Britain. In Poland, it was first noted in 1923–1924 and spread rapidly in 2007–2008, affecting up to 58.3% of sea trout in the Rega river [9]. Fish with UDN have skin lesions that are quickly infected with *Saprolegnia*, giving the affected fish the appearance of being covered in slimy white pustules [1,7]. Infection is favored by the presence of impurities, water temperatures that are too low, and the weakening of fish during spawning migration. High intercellular levels of heavy metals can be toxic, resulting in alterations in the intercellular protein machinery via the denaturation of enzymes or the generation of reactive oxygen species (ROS) [10]. The relationship between metal concentrations and the pathological state of different fish species has been previously studied by others [11,12,13].

Besides the aforementioned ecological implications, heavy metals in fish can also pose a significant risk to human health when consumed in amounts exceeding safe consumption levels. They may accumulate in different tissues, causing both chronic and acute health problems. The main metal threats to human health have been mainly associated with Hg, Cd, and Pb exposure [14]. They are absorbed by humans through diet, and show no beneficial effects on health. Some methods have been developed for the assessment of human health risks from carcinogenic and non-carcinogenic metals associated with fish consumption. To evaluate potential non-carcinogenic health risks, target hazard quotient (THQ) and total THQ are used [15].

The aim of this study was to determine whether there is any relationship between the content of the heavy metals, lead (Pb), cadmium (Cd), and mercury (Hg) in selected organs of sea trout and the occurrence of external symptoms of UDN. Moreover, for the sake of consumer health, another aim of the study was to assess the dietary uptake of Pb, Cd, and Hg from the muscles of healthy sea trout and to establish whether Cd, Hg, and Pb concentrations were below the maximum limits, as set by European legislation [16].

## 2. Materials and Methods

### 2.1. Study Area

**Figure 1:**
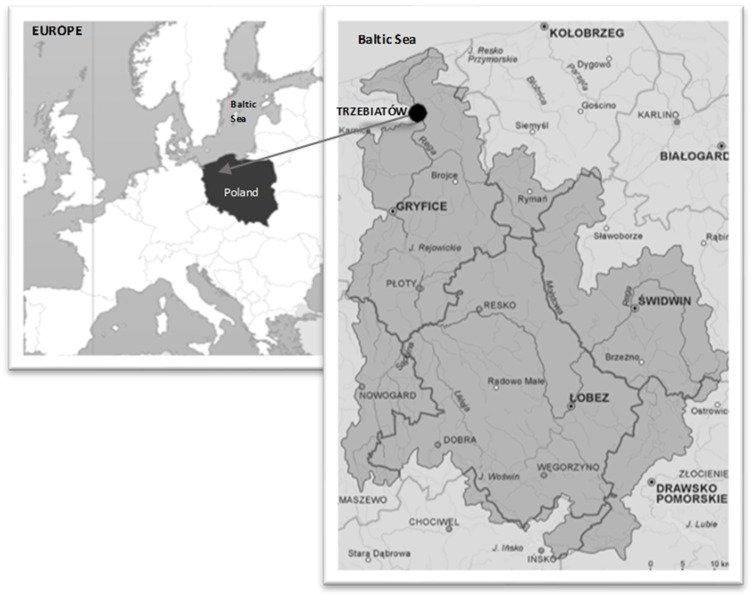
Location of the study area on the river Rega, north-western Poland.

The study was carried out on the section of the river Rega that flows through the town of Trzebiatów, north-western Poland, at the field station of the Polish Angling Association (54°02′56.4″ N 15°16′17.7″ E) (Figure 1). The river Rega is Poland’s third-longest river (167.8 km), flowing into the Baltic Sea. It is an extremely important ecological passageway for the migration of spawning salmonids, as well as a perfect habitat for non-migratory salmonids species [17,18]. The river Rega is of great significance as one of the few Polish rivers to which salmons migrate to spawn. However, numerous hydro-engineering structures make it difficult, or even impossible, for fish to reach their spawning grounds, which are located in the upper course of the river.

### 2.2. Materials

Male (m) and female (f) sea trout were caught in the river Rega during the spawning period from mid-October to early December 2013. Unfortunately, during the six selected fishing days, no healthy males were found among the caught fish (86 individuals). The fish were delivered to the laboratory on the catching day and their weight and size were measured (Table 1). Depending on whether any external UDN-like symptoms were present or not, all the fish were divided into two groups: UDN—fish with UDN symptoms (unhealthy), and H—fish without UDN symptoms (healthy). The infection patterns were as described by Ciepliński et al. [6] who showed differences in the external symptoms of infection in males and females resulting from sexual dimorphism in the skin structure of sexually mature sea trout.

Among all the fish caught, 20 males (m) and 20 females (f) with UDN-like symptoms (UDN) and 20 females without symptoms (H) were selected for the study. The selected fish were of similar dimensions. Sex of the fish was determined by direct observation of gonads. Fish autopsy was performed using stainless steel tools to prevent contamination of the samples. Samples of the dorsal muscle and selected organs, including liver, kidneys, gonads, and gills were taken from each fish, rinsed in distilled water and stored in clean, labelled polyethylene bags at −20 °C until the time of metal analysis.

The standard reference material (SRM) used in this study was DORM-4 (fish protein homogenate for trace metals), certified by the National Research Council of Canada.

### 2.3. Sample Preparation

To prepare the samples, the examined material was first homogenised in an agate mortar and then weighed. To prepare analytical samples for Pb and Cd determination, 1 g wet weight (ww) of the selected organs or 2 g of muscles were weighed in Teflon^®^ vessels with an accuracy of ±0.001 g. In each batch (12 samples), three samples were analysed twice and one in triplicate. Digestion was carried out in a microwave oven CEM MDS 2000 using 5 mL of EMSURE^®^, 65% nitric acid (Merck KGaA, Darmstadt, Germany). The digestion process proceeded in three steps under the following conditions: (1) 50 PSI for 5 min; (2) 75 PSI for 5 min; (3) 85 PSI for 10 min. After digestion, the samples were transferred into a weighed polyethylene bottle and filled up with deionised water to 20 g (±0.001 g). In order to evaluate the accuracy of the methods used, besides the tested material, one sample (0.15 g) of certified material (DORM-4) and two blank samples (5 mL of 65% HNO_3_) were subjected to mineralization in every batch.

In case of mercury determination, 1 g ww of fish organs or 5 g ww of muscles (±0.001 g) were placed in conical flasks and digested in a mixture of concentrated HNO_3_ and HClO_4_ (*v*/*v*, 4:1) using an incubator at 70 °C for 48 h. In parallel, blanks and reference material (DORM-4) were prepared similarly to the test material.

Moisture content in the examined material was analysed following the AOAC standard method 950.46 (forced-air oven drying) [19]. Dry matter content was calculated as the difference between wet weight and moisture content in organs and expressed in percentages (Table 2).

### 2.4. Determination of Lead (Pb), Cadmium (Cd), and Mercury (Hg)

Lead and cadmium were determined using graphite furnace atomic absorption spectrometry (GF-AAS, Perkin Elmer ZL 4110) at the following wavelengths: Pb 220.353 nm, Cd 228.802 nm. The system was equipped with Zeeman background correction, an argon-powered furnace, and a measurement recording system. Mercury was analysed after reduction with SnCl_2_, at a wavelength of 253.7 nm, by cold vapour atomic absorption spectrometry (CV-AAS) in a Bacharach Coleman MAS 50D apparatus.

Quantification of the elements was performed on the basis of external standard curves. Calibrations were performed using five metal concentrations (µg/L) in the following ranges: 1.0–50 (Pb), 0.20–2.5 (Cd), and 0.50–10.0 (Hg). For six different calibration curves, the obtained coefficients of determination (R2) were higher than 0.9993. No outlying measurements >3 times the standard error of the calibration function were found. The limits of detection (LOD) were determined and calculated using calibration curve data and were as follows (µg/L): Pb—1.06, Cd—0.067, Hg—0.3. In all the analyses, metal concentrations in blanks were below the LODs. The analyses were duplicated to check the reproducibility of the results. Relative standard deviations (RSD) among replicates were all below 10%. Based on the metal determination in the reference material (DORM-4), the following recoveries (%) and RSD (%) for the applied methods were obtained: Pb—95.7; 3.2, Cd—95.9; 4.0, Hg—97.6; 3.7. All determinations were performed in triple replications. Metal concentrations are presented in µg/g ww throughout the manuscript.

### 2.5. Consumer Health Risk Assessment

The results of the laboratory analyses were compared with the highest permissible metal levels in fish and fish products, specified in the amended EU Commission Regulation (EC 1881/2006) [16]. Moreover, this study assessed the possible health effects of consuming the muscles of healthy sea trout (H). For this purpose, the following risk factors established by the U.S. Environmental Protection Agency [15,20] were calculated: EDI (estimated daily intake in µg/kg body weight (bw)), THQ (target hazard quotient). and HI (hazard index). The factors were estimated using the following Equations (1–3):

(1) $$\mathrm{EDI}=\frac{C\times \mathrm{IR}}{\mathrm{BW}}\;$$

where: C—metal concentration in sea trout muscles (fH) (μg/g ww); IR—daily ingestion rate (g/d); BW—body weight (kg);

(2) $$\mathrm{THQ}=\frac{\mathrm{EF}\times \mathrm{ED}\times \mathrm{MS}\times C}{\mathrm{RfD}\times \mathrm{BW}\times \mathrm{AT}}\times 10^{-3}$$

where: EF—exposure frequency (365 days/year); ED—exposure duration (70 years); MS—food ingestion rate (g/day); C—metal concentration in sea trout muscles (fH) (μg/g ww); RfD—oral reference dose (mg/kg/day) for Pb (0.0036), Cd (0.001), and Hg (1.0 × 10^−4^); BW—body weight (kg); AT—average exposure time to non-carcinogens (365 days/year × ED).

The hazard index (HI) was calculated as a sum of THQ values (Equation (3)):

(3) $$\mathrm{HI}=\overset{n}{\underset{i=1}{\sum }}\mathrm{THQ}$$

The HI was used in this study to describe the cumulative non-carcinogenic effect. A result of less than one signified non-obvious risk. Conversely, an exposed human population of concern will experience health risk if the dose is equal to or greater than the RfD.

For all the above calculations (Equations (1)–(3)), the following assumptions were made: body weight of an adult—70 kg; recommended fish consumption—two servings of about 150 g per week [21]; mean concentrations of metals in fish muscles (fH).

The estimated daily intake (EDI) was compared with the BMDL (benchmark dose lower confidence limit) determined for the effects of Pb in adults on the cardiovascular system (BMDL_01_ 1.5 µg/kg bw/day) and nefrotoxicity (BMDL_10_ of 1.5 and 0.63 µg/kg bw/day) as potential critical adverse health effects [21]. Weekly intake (EDI × 7) was related to the TWI (tolerable weekly intake) of 2.5 μg/kg bw for Cd [22] and 1.3 µg/kg bw for methylmercury (Me-Hg) [23]. In order to assess Me-Hg content in sea trout muscles, the results for total mercury (T-Hg) were converted using factors of 1 for methylmercury and 0.2 for inorganic mercury [23].

### 2.6. Statistical Analysis

All statistical calculations were performed using Statistica ver. 10 software (Statsoft Inc., Tulsa, OK, USA). Post-hoc Duncan’s test was used to determine statistically significant differences in the concentrations of Pb, Cd, and Hg between the selected organs of sea trout with respect to fish sex and health status. Differences with a significance of *p* ≤ 0.05 were considered statistically significant.

## 3. Results and Discussion

### 3.1. Fish Size and Dry Mass Content in Organs and Tissues of Sea Trout

**Figure 2:**
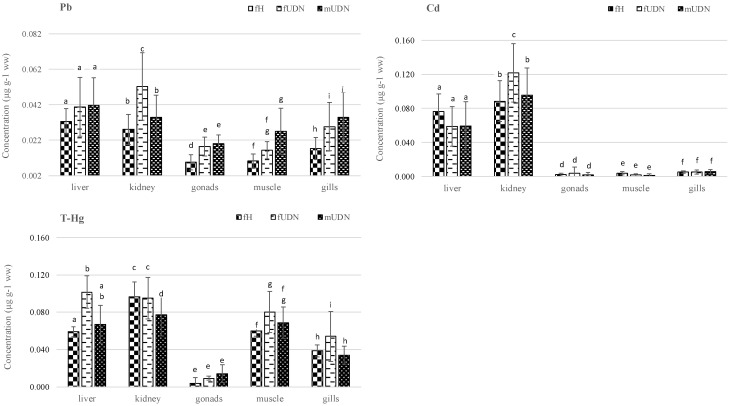
Differences in metal (Pb, Cd, Hg) content in organs of sea trout depending on health status. fH—females without symptoms of disease; fUDN—females with symptoms of disease; mUDN—males with symptoms of disease. ^a–h^ Different letters for the same organs indicate significant inter-group differences in metal concentration (Duncan’s test comparison of means, *p* < 0.05).

The morphometric data of the studied sea trout are presented in Table 1. The statistical analysis indicated that the sea trout with UDN symptoms (fUDN, mUDN) were smaller than the healthy fish (fH). Both males and females with symptoms of the disease had kidneys of significantly lower dry weight compared with the healthy individuals (Table 2). Dry matter is an indicator of the amount of nutrients available to the animal. The percentage of dry matter (dm) in the other organs was comparable between the groups of healthy (fH) and unhealthy (fUDN) females. The males had significantly lower dry matter content (%) in the gonads and gills compared with the females (fH; fUDN). Given the differences in metal content in fish organs (Table 3, Figure 2), it may be useful to convert the results to dry matter to investigate the effect of organ moisture on these differences.

### 3.2. Metal Concentrations in Fish Organs Depending on Sex and Health Condition

The distribution of heavy metals (Pb, Cd, Hg) in the organs and muscles of the studied sea trout in relation to the fishes’ health condition (fH, fUDN, mUDN) is presented in Table 3 and Figure 2.

The metal levels in fish usually follow the following ranking: Pb > Cd > Hg [24]. In this study, when the mean metal levels were considered without taking into account fish health status and sex, the following quantitative relationships were generally found: liver Hg > Cd > Pb; kidneys Cd > Hg > Pb; gonads Pb > Hg > Cd; muscles and gills Hg > Pb > Cd. The same accumulation order for Pb and Cd was observed in the liver, kidneys, and muscle tissues of the Atlantic salmon [25]. Another study reported a reverse relationship between Pb and Cd accumulation in the liver and gills: Pb > Cd and Cd > Pb, respectively [26]. Considering all the organs, the highest metal levels were detected in the livers and kidneys of the sea trout. The largest amounts of Cd and Hg were found in the kidneys, while the lowest levels of all the analysed metals were recorded in gonads. The results obtained in this study are consistent with other studies [27], which indicate that kidneys often contain high levels of metals due to their excretory function and that gonads are less contaminated with heavy metals. In general, the distribution of heavy metals in the organs of sea trout can be presented in the following way: Pb: liver > kidneys > gills > muscles > gonads; Cd: kidney > liver > gills > muscles > gonads; Hg: kidney > muscles > liver > gills > gonads. The presence of the highest Cd concentrations in the kidneys were also reported by other researchers [28]. Another study of brown trout organs [29] showed a comparable pattern of accumulation for Cd (liver > gills > muscles), but different patterns in the case of Pb (gills > liver > muscles) and Hg (liver > gills > muscles). The mercury content in the organs of both of the analysed sea trout female groups (fH, fUDN) was generally as follows: kidney > liver > muscle > gills > gonads. Moreover, it was found that Hg concentrations were significantly higher in the liver, muscles, and gills of unhealthy female fish (fUDN) (*p* < 0.05) than in females without UDN symptoms (fH) (Figure 2). In a similar study [11], but in another fish species (*Limanda limanda*), higher Hg content was also found in the muscles of diseased specimens (ulcers, symptoms of lymphocytosis or papillomas) compared to healthy specimens.

All of the examined organs of the diseased individuals (fUDN, mUDN) had higher mean concentrations of Pb compared with the organs of healthy fish (fH), but not all of the differences were statistically significant (Figure 2). It was also found that diseased sea trout fUDN, mUDN) had significantly higher Cd and Hg contents in their kidneys compared with their livers (Table 3). The study cited above [11] indicated no statistically significant differences in the content of Pb and Cd in organs between healthy and diseased individuals. However, other researchers [12] found significantly higher Pb content in the muscles and Cd in the muscles and livers of diseased Boulogne dabs compared to healthy fish. In the groups of female fish (fH, fUDN), the Pb concentration in the muscles was significantly lower than in the liver and kidneys. In the case of diseased males (mUDN), lead content in the muscles was comparable to Pb content in the other organs (Table 3). The level of cadmium in the muscles of all the examined fish was significantly lower than in the livers and kidneys and comparable to its concentration in the gonads and gills. The low cadmium content in the gills may indicate that the fish ingested this metal mainly from food [29]. The level of Hg in the muscles was significantly higher than in the gonads and gills. In the case of fish with symptoms of the disease (UDN), the Hg content in the muscles was comparable to its content in the liver (fUDN, mUDN) and kidneys (mUDN). The role of fish sex in mercury bioaccumulation was presented and explained in the review by Madenjiana et al. [30]. According to authors, male fish not only ingest but also eliminate Hg at a higher rate than females, which is most likely due to some androgens, such as testosterone and 11-ketotestosterone, enhancing the Hg elimination rate in males. The above conclusion is in line with our results, which, apart from the gonads, indicate a lower THg content in the organs of male sea trout (mUDN) compared to females (fUDN). However, these differences were statistically significant only in the case of gills and muscles (Table 3). Apart from the aforementioned cases, the statistical analysis did not indicate any clear relationship between Pb, Cd and Hg concentrations and the occurrence of UDN-like symptoms in sea trout.

Compared with other tissues, fish muscles usually contain low levels of metals but are often examined due to their importance for human safety [31]. The contents of heavy metals in the analysed sea trout muscles (in wet weight) ranged as follows: lead 0.006–0.053 µg/g, cadmium <LOD-0.006 µg/g, and mercury 0.048–0.131 µg/g (Table 3). The study cited above [31] showed that even if the concentration of metals in the liver, kidneys or gills is high, their level in muscles may be undetectable. This also applies to the present study, which shows that the concentration of Cd in muscles was as much as 61 times lower than in the liver. The content of Hg in the muscles of all the groups of sea trout was significantly higher than in the gills and gonads, but in the case of fH and mUDN, it was comparable with the Hg content in the liver (Table 3). Similar observations were made by other authors [24], who indicated that mercury is probably the only metal whose concentrations in muscles may exceed those in other organs. Regarding the sex of the sea trout, higher Hg accumulation was observed in the muscles of the females (fH, fUDN) than in the males (mUDN). A study on Hg levels in Amazonian fish [32] showed that sex is not the main driver of Hg bioaccumulation. In eight studies, males had higher concentrations in muscles than females, whereas in seven other studies, the opposite was observed. Moreover, the authors concluded that neither the surveillance of environmental pollution nor current food advice based on muscle T-Hg needed to be changed because of fish sex.

### 3.3. Health Risk Assessment for Fish Consumption

The concentrations (µg/g ww) of Pb (0.006–0.021), Cd (<LOD-0.005), and Hg (0.048–0.096) in the muscles of healthy sea trout (fH) did not raise any objections as to the maximum levels in food (MRL), which are as follows: Pb—0.3 µg/g, Cd—0.05 µg/g, Hg—0.5 µg/g [16]. Higher ranges of Pb, Cd and Hg content (µg/g ww) were found in fish from the Adriatic Sea: <LOD–1.18, 0.01–0.05 and 0.07–1.56, respectively [33]. Other authors [34] reported higher concentration of Pb (0.051 µg/g ww) and much lower of Cd (3.8 × 10^−4^ µg/g ww) in the muscles of rainbow trout. A much higher content of Pb (0.158 µg/g ww), a slightly higher content of Cd (0.012 µg/g ww), and a comparable amount of Hg (0.055 µg/g ww) were detected in the muscles of *Salmo trutta* (m. *fario*) [35]. A concentration of Hg (0.1 µg/g) that was about 1.7 times higher was recorded in commercial fish and other seafood [36] and in the muscles of brown trout [37]. The above concentrations, however, correspond to the highest value obtained in this study (Table 3).

According to the Statistical Yearbook of the Republic of Poland (2019) [38], the average monthly consumption of fish and seafood in Poland is 0.28 kg per person, which is 70 g/week and 9.2 g/day. In this study, Pb, Cd, and Hg uptake from the muscles of healthy sea trout (fH) was assessed based on the portion recommended by most European Food-Based Dietary Guidelines, which is (a minimum of) two servings (of about 150 g each) of fish per week (42.9 g daily) for older children, adolescents, and adults [21,39]. When assessing the risk to human health from potentially harmful chemicals in food, it is crucial to keep in mind that the dietary intake of such substances must remain within set safety margins. In our study, the estimated daily intake (EDI) of Pb was 0.006 µg/kg bw/d, which is only 0.4% of BMDL_01_ and 1.0% of BMDL_10_, which means no cardiovascular or nephrotoxicity risk for adults. The mean EDI for cadmium was three times lower than that for Pb. Expressed as weekly intake (EWI), this value was 0.014 µg/kg bw and accounted for only 0.56% of TWI (Table 4). Other authors reported significantly higher Pb levels in rainbow trout muscles, which also resulted in a greater coverage of BMDL_10_ (9.73%) and BMDL_01_ (24.5%) [40].

The estimated tolerable daily intake (ETDI) of Pb and Cd studied by others [41] was also lower than the PTDI (provisional tolerable daily intake). The uptake of Pb and Cd from fish against the background of the total dietary exposure of Polish students accounted for 6.9–30% and 18–67% of PTWI (provisional tolerable weekly intake), respectively [42]. The EDI values for Pb, Cd, and Hg related to the consumption of *Clupeonella cultriventris* were also safe for consumers taking into account the respective RfDs [43]. In the present study, the highest EDI was recorded for total mercury (0.029–0.058 µg/kg bw), with as much as 0.049 µg/kg bw for Me-Hg, which accounts for 26.1% of the TWI (Table 4). It has been shown that fish consumption may be an important route of exposure to mercury in humans. Some authors [44] pointed out that fish essentially accounts for the majority of Hg uptake. They recorded Hg uptake from fish in a wider range of 0.003–0.206 µg/kg bw/day but obtained a comparable coverage of PTWI. Our results are in line with the opinion of the EFSA [23], which indicates that in adults, the mean dietary exposure to methylmercury does not exceed the TWI. High Hg intake through fish consumption amounting to 90% of PTWI has been observed in riverside inhabitants of the Urrá reservoir, Colombia [45]. According to EFSA [23], when consuming species with high methylmercury content, the TWI is reached after eating a few servings only (<1–2). Paraphrasing others [44], we concluded that although our estimates were in line with the recommended TWI, emphasis should be placed on the fact that Hg toxicity is related to the chemical form present in the matrix, with organic molecules such as methylmercury being the most toxic. Our calculations show that a safe amount of sea trout should not exceed 1.8 kg weekly (Table 4). Much higher results were obtained for lead (4.2–10.7 kg daily) and cadmium (56.3 kg weekly). Although fish and seafood are the primary sources of methylmercury for consumers [23], its intake from other foods should also be considered. Moreover, more frequent consumption of fish than was assumed in this study (high levels of consumption) could also pose a threat to health. Given a weekly consumption of sea trout six times greater than assumed, the consumer would ingest Me-Hg in an amount equal to 100% of the TWI (1800 g, i.e., 257.1 g/day). Therefore, increased fish intake can pose a risk, especially in populations with traditionally high fish and seafood consumption, as they are particularly susceptible to Hg poisoning [46].

THQ as the integrated risk index compares the ingested amount of a contaminant with a standard reference dose [15]. The results showed that THQ values for individual metals as well as HI for combined metals were lower than 1 as a result of consuming the assumed portion of sea trout (Table 4). The above observations were identical to those made by other authors [36,41,43,47]. The average THQ value for Hg was significantly higher than that for Pb and Cd, with a maximum of up to 0.582. THQ < 1 signifies that the level of exposure is lower than the reference dose, which means that a daily exposure at this level is not likely to cause any negative health effects over a lifetime in a human population [15,44]. In Adriatic fish, THQ ranges of 0.01–0.04 and 0.002–0.18 were reported for Cd and Pb, respectively [33], indicating that the health risk associated with the consumption of fish was insignificant. Conversely, mercury THQ values ranging from 0.08 to 1.87 were of concern. The average THQ for T-Hg had a significant impact (98.9%) on the hazard index (HI) value (Table 4). A higher HI index (close to 1) was observed in bluefin tuna and mackerel, which was also due to their Hg content [47]. The authors of the research pointed out that these two fish species should be consumed in moderation, especially by pregnant women. In another study [43], out of eight analysed metals, Cd was found to make the highest contribution to the HI value. Taking into account the above results, the consumption of healthy sea trout muscles is not of significant concern. However, an intake six times larger than the daily portion assumed in this study (42.9 g) would exceed the safe TWI value for Hg.

## 4. Conclusions

The levels of Pb, Cd, and Hg in some organs of sea trout depended on their health status. A significantly higher amount of Pb was observed in the gonads, gills, and muscles, of Cd in the kidneys, and of Hg in the muscles of sea trout showing external symptoms of UDN-like disease. Although some significant relationships were observed between the content of Pb, Cd, and Hg in organs and the occurrence of UDN-like symptoms in sea trout, further detailed studies are advisable.

The muscles of healthy sea trout contained Pb, Cd, and Hg at levels that were safe for consumers. Moreover, the calculated values of THQ and HI, as well as the recorded heavy metal intake as compared to BMDLs and TWI, do not raise any health concerns for an adult consumer of healthy sea trout muscles. However, health risk related to Hg uptake for consumers of higher levels cannot be excluded.

## Figures and Tables

**Table 1:** Morphometric data of sea trout (*Salmo trutta* m. *trutta* L.) from the river Rega.

Sea Trout	*n*	Weight (g)	Length (cm) ^1^
		$\bar{x}$ ± SD (Min–Max)	$\bar{x}$ ± SD (Min–Max)
female (f)			
healthy (fH)	20	1779.0 ^a^ ± 368.9 (1385.0–2615.0)	58.8 ^a^ ± 3.3 (55.5–65.5)
unhealthy (fUDN)	20	1769.5 ^a^ ± 219.5 (1480.0–2140.0)	56.6 ^b^ ± 3.5 (52.0–64.0)
male (m) healthy(mH)	0 ^2^		
unhealthy (mUDN)	20	1724.0 ^a^ ± 444.0 (1190.0–2540.0)	55.8 ^b^ ± 4.0 (50.0–61.0)
all groups	60	1756.5 ^1^ ± 363.6 (1190.0–2615.0)	56.9 ± 3.8 (50.0–65.5)

$\bar{x}$—mean; ±SD—standard deviation; ^1^ length from the tip of the snout to the tip of the longer lobe of the caudal fin; f—female; m—male; health status: H—healthy (without UDN symptoms); UDN—with UDN symptoms; ^2^ healthy males (mH) could not be obtained on the selected fishing days; ^a,b^ values with different letters in column differ significantly (Duncan’s test, *p* < 0.05).

**Table 2:** Dry matter of organs and tissues of sea trout (*Salmo trutta* m. *trutta* L.).

Organ	Dry Matter (%)		
	fH	fUDN	mUDN
	$\bar{x}$ ± SD	$\bar{x}$ ± SD	$\bar{x}$ ± SD
liver	19.7 ^a^ ± 0.9	18.8 ^a^ ± 1.6	21.6 ^b^ ± 3.1
kidney	19.0 ^a^ ± 2.0	16.1 ^b^ ± 2.3	17.1 ^b^ ± 1.7
gonads	37.2 ^a^ ± 2.8	36.0 ^a^ ± 2.0	28.1 ^b^ ± 2.3
muscles	21.0 ^a^ ± 1.8	21.1 ^a^ ± 1.6	20.8 ^a^ ± 1.2
gills	17.0 ^a^ ± 2.1	17.7 ^a^ ± 1.7	15.6 ^b^ ± 1.9

$\bar{x}$—mean; ±SD—standard deviation; *n* = 20; f—female; m—male; health status: H—healthy (without UDN symptoms); UDN—with UDN symptoms; ^a,b^ values with different letters in the same row differ significantly (*p* < 0.05).

**Table 3:** Mean contents of Pb, Cd, and Hg in the organs of sea trout (µg/g ww).

Group	Metal	Liver	Kidney	Gonads	Gills	Muscle
		$\bar{x}$ (Min–Max)				
fH	Pb	0.033 ^a^	0.028 ^a,c^	0.009 ^b^	0.017 ^b,c^	0.010 ^b^
		(0.024–0.046)	(0.019–0.039)	(0.005–0.013)	(0.009–0.034)	(0.006–0.021)
	Cd	0.076 ^a^	0.089 ^a^	0.002 ^b^	0.005 ^b^	0.004 ^b^
		(0.057–0.096)	(0.055–0.117)	(n.d.–0.005)	(0.002–0.011)	(n.d.–0.005)
	T-Hg	0.059 ^a^	0.096 ^b^	0.004 ^b^	0.039 ^d^	0.060 ^a^
		(0.051–0.069)	(0.077–0.135)	(n.d.–0.008)	(0.032–0.049)	(0.048–0.095)
	Me-Hg					0.050
						(0.040–0.079)
fUDN	Pb	0.04 ^a,c^	0.052 ^a^	0.018 ^b^	0.030 ^b,c^	0.016 ^b^
		(0.029–0.061)	(0.035–0.077)	(0.011–0.025)	(0.024–0.049)	(0.007–0.035)
	Cd	0.059 ^a^	0.122 ^b^	0.004 ^c^	0.005 ^c^	0.002 ^c^
		(0.042–0.113)	(0.084–0.141)	(n.d.–0.023)	(0.002–0.015)	(n.d.–0.005)
	T-Hg	0.102 ^a^	0.095 ^a^	0.009 ^c^	0.054 ^d^	0.080 ^c^
		(0.070–0.195)	(0.077–0.182)	(0.002–0.017)	(0.029–0.072)	(0.056–0.131)
	Me-Hg					0.067
						(0.047–0.10)
mUDN	Pb	0.042 ^a^	0.035 ^a^	0.020 ^b^	0.029 ^a,b^	0.027 ^a,b^
		(0.023–0.069)	(0.024–0.056)	(0.015–0.041)	(0.017–0.059)	(0.016–0.053)
	Cd	0.060 ^a^	0.096 ^b^	0.002 ^c^	0.006 ^c^	0.002 ^c^
		(0.047–0.135)	(0.065–0.163)	(n.d.–0.008)	(0.002–0.009)	(n.d.–0.006)
	T-Hg	0.067 ^a^	0.077 ^a^	0.014 ^b^	0.034 ^c^	0.069 ^a^
		(0.040–0.093)	(0.046–0.122)	(n.d.–0.028)	(0.023–0.050)	(0.049–0.102)
	Me-Hg					0.058
						(0.04.085)

$\bar{x}$—mean; ww—wet weight; f—female; m—male; health status: H—healthy (without UDN symptoms); UDN—with UDN symptoms; T-Hg—total mercury; Me-Hg—mercury in methyl form; n.d.—not detected; ^a–d^ values with different letters in the same row within the group differ significantly (Duncan’s test, *p* < 0.05).

**Table 4:** Potential human health risk assessment for adults ^1^.

	Pb	Cd	T-Hg	Me-Hg
	Mean (Min–Max)			
EDI (µg/kg bw)	0.006	0.002	0.037	0.031
	(0.004–0.013)	(n.d.–0.003)	(0.029–0.058)	(0.025–0.049)
EWI (µg/kg bw)	0.042	0.014	0.259	0.215
	(0.028–0.091)	(n.d.–0.021)	(0.203–0.406)	(0.172–0.340)
Percentage of				
BMDL_01_	0.4 (0.3–0.9)			
BMDL_10_	1.0 (0.6–2.1)			
TWI		0.56 (n.d.–0.84)		16.5 (13.2–26.1)
MPF (kg) ^2^				
Daily	10.7			
	4.2			
Weekly		56.3		1.8
THQ	0.002	0.002	0.368	0.306
	(0.001–0.004)	(n.d.–0.003)	(0.294–0.582)	(0.294–0.485)
HI	0.372			
%THQ ^3^	0.54	0.54	98.92	

^1^ An adult—70 kg bm; ^2^ MPF- Maximum portion of fish covering 100% BMDLs or TWI; ^3^ Mean percentage contribution of THQs in HI; n.d.—not detected; T-Hg—total Hg; Me-Hg –methylmercury; EDI-estimated daily intake; EWI-estimated weekly intake (EDI×7); BMDL-Benchmark Dose Lower Confidence Limit; TWI-Tolerable Weekly Intake; THQ-hazard quotient; HI-hazard index.

## Data Availability

The data that support the findings of this study are available from the corresponding author, upon reasonable request.

## References

[1] Svobodová Z., Lloyd R., Máchová J., Vykusová B. (1993). Water Quality and Fish Health.

[2] Authman M.M.N., Zaki M.S., Khallaf E.A., Abbas H.H. (2015). Use of fish as bio-indicator of the effects of heavy metals pollution. J. Aquac. Res. Dev..

[3] Garai P., Banerjee P., Mondal P., Saha N.C. (2021). Effect of heavy metals on fishes: Toxicity and bioaccumulation. J. Clin. Toxicol..

[4] Kallio-Nyberg I., Saura A., Ahlfors P. (2002). Sea migration pattern of two sea trout (*Salmo trutta*) stocks released into the Gulf of Finland. Ann. Zool. Fen..

[5] ICES (2019). Sea Trout (Salmo trutta) in Subdivisions 22–32 (Baltic Sea).

[6] Ciepliński M., Kasprzak M., Grandtke M., Giertych M.J., Steliga A. (2018). Pattern of secondary infection with *Saprolegnia* spp. in wild spawners of UDN-affected sea trout *Salmo trutta* m. *trutta* (L.), the Słupia River, N Poland. Oceanol. Hydrobiol. Stud..

[7] Tkachenko H., Kurhalyuk N., Pałczyńska K. (2011). Responses of antioxidant status in the gills of brown trout (*Salmo trutta* m. *trutta* L.) with ulcerative dermal necrosis. Balt. Cost. Zone. J. Ecol. Prot. Coastline.

[8] Roberts R.J. (1993). Ulcerative dermal necrosis (UDN) in wild salmonids. Fish. Res..

[9] Bartel R., Bernaś R., Grudniewska J., Jesiołowski M., Kacperska B., Marczyński A., Pazda R., Pender R., Połomski S., Skóra M. (2009). Furunculosis in salmon (*Salmo salar*) and sea trout (*Salmo trutta trutta*) in Poland in 2007 and 2008. Komun. Ryb..

[10] Pourahmad J., O’Brien P.J. (2000). A comparison of hepatocyte cytotoxic mechanism for Cu^2+^ and Cd^2+^. Toxicology.

[11] Protasowicki M. (1991). Preliminary studies on selected elements in organs of dab *Limanda limanda* and their relation to fish disease state. Mar. Ecol. Prog. Ser..

[12] Henry F., Amara R., Courcot L., Lacouture D., Bertho M.L. (2004). Heavy metals in four fish species from the French coast of the Eastern English Channel and Southern Bight of the North Sea. Environ. Int..

[13] Rodjuk G.N., Chukalova N.N., Shenderyuk V.V., Bakholdina L.P., Chernysheva N.L., Sayadov S.O. (2012). Prevalence of skin ulceration in cod (Gadus morhua callarias L.) under anthropogenic contamination in the southeastern part of the Baltic Sea. Inland Water Biol..

[14] Järup L. (2003). Hazards of heavy metal contamination. Br. Med. Bull..

[15] US EPA (2015). Quantitative Risk Assessment Calculations.

[16] European Comission (2006). Commission Regulation (EC) No 1881/2006. Off. J. Eur. Union.

[17] Bartel R. (2001). Return of salmon back to Polish waters. Ecohydrol. Hydrobiol..

[18] Cedro B. (2007). Evolution of the River Rega valley near Łobez in late Pleistocene and early Holocene. Geochronometria.

[19] AOAC (2000). Official Methods of Analysis.

[20] USEPA (2000). Risk-Based Concentration Table.

[21] European Food Safety Authority (EFSA) (2012). Lead dietary exposure in the European population. EFSA J..

[22] European Food Safety Authority (EFSA) (2012). Cadmium dietary exposure in the European population. EFSA J..

[23] European Food Safety Authority (EFSA) (2012). Panel on Contaminants in the Food Chain (CONTAM). Scientific Opinion on the risk for public health related to the presence of mercury and methylmercury in food. EFSA J..

[24] Jezierska B., Witeska M., Twardowska I., Allen H.E., Häggblom M.M., Stefaniak S. (2006). The metal uptake and accumulation in fish living in polluted waters. Soil and Water Pollution Monitoring, Protection and Remediation.

[25] Sauliute G., Svecevicius G. (2017). Heavy metals (Zn, Cu, Ni, Cr, Pb, Cd) in water and body tissues of young atlantic salmon *Salmo salar* in two rivers of different pollution level: A comparison with fish condition parameters. Fresenius Environ. Bull..

[26] Vinodhini R., Narayanan M. (2008). Bioaccumulation of heavy metals in organs of fresh water fish Cyprinus carpio (*Common carp*). Int. J. Environ. Sci. Tech..

[27] Has-Schön E., Bogut I., Strelec I. (2006). Heavy metal profile in five fish species included in human diet, domiciled in the end flow of river Neretva (Croatia). Arch. Environ. Contam. Toxicol..

[28] Linde A.R., Sánchez-Galán S., Klein D., García-Vázquez E., Summer K.H. (1999). Metallothionein and heavy metals in brown trout (*Salmo trutta*) and European eel (*Anguilla anguilla*): A comparative study. Ecotoxicol. Environ. Saf..

[29] Can E., Yabanli M., Kehayias G., Aksu Ö., Kocabas M., Demir V., Kayim M., Kutluyer F., Şeker S. (2012). Determination of bioaccumulation of heavy metals and selenium in tissues of brown trout *Salmo trutta macrostigma* (Dume´ril, 1858) from Munzur Stream, Tunceli, Turkey. Bull. Environ. Contam. Toxicol..

[30] Madenjian C.P., Rediske R.R., Krabbenhoft D.P., Stapanian M.A., Chernyak S.M., O’Keefe J.P. (2016). Sex differences in contaminant concentrations of fish: A synthesis. Biol. Sex Differ..

[31] Perkins E.J., Griffin B., Hobbs M., Gollon J., Wolford L., Schlenk D. (1997). Sexual differences in mortality and sublethal stress in channel catfish following a 10 week exposure to copper sulfate. Aquat. Toxicol..

[32] Bastos W.R., Dórea J.G., Bernardi J.V.E., Manzatto A.G., Mussy M.H., Lauthartte L.C., Lacerda L.D., Malm O. (2016). Sex-related mercury bioaccumulation in fish from the Madeira River, Amazon. Environ. Res..

[33] Storelli M.M. (2008). Potential human health risks from metals (Hg, Cd, and Pb) and polychlorinated biphenyls (PCBs) via seafood consumption: Estimation of target hazard quotients (THQs) and toxic equivalents (TEQs). Food Chem. Toxicol..

[34] Varol M., Sünbü M. (2017). R Comparison of heavy metal levels of farmed and escaped farmed rainbow trout and health risk assessment associated with their consumption. Environ. Sci. Pollut. Res..

[35] Makovský J., Spurný P., Mareš J., Hedbávný J., Vítek T. (2010). Heavy metal pollution of ecosystem within the middle course of the Jihlava River. Acta Univ. Agric. et Silvic. Mendel. Brun..

[36] Goldman L.R., Shannon M.W. (2001). and the Committee on Environmental Health. Technical Report: Mercury in the Environment: Implications for Pediatricians. Pediatrics.

[37] Falandysz J., Chwir A., Wyrzykowska B. (2000). Total mercury contamination of some fish species in the firth of Vistula and the lower Vistula river, Poland. Pol. J. Environ. Stud..

[38] Bielak R., Borek D., Głowacka-Smolis K., Gustyn J., Kozera A., Kozłowska J., Marikin M., Morytz-Balska E., Rybak-Nguyen E., Safader M. (2019). Statistical Yearbook of the Republic of Poland (GUS).

[39] European Food Safety Authority (EFSA) (2015). Scientific Committee Statement on the benefits of fish/seafood consumption compared to the risks of methylmercury in fish/seafood. EFSA J..

[40] Staszowska A., Skałecki P., Florek M., Litwińczuk A. (2013). Influence of fish species and environment on lead content and estimation of lead uptake from muscle tissue. Zywn. -Nauk. Technol. JA.

[41] Kumar A., Kumar A., Jha S.K. (2020). Human health risk assessment of heavy metals in major carp (*Labeo rohita*) of Mahananda river in Northern India. Emer. Life Sci. Res..

[42] Marzec Z., Koch W., Marzec A., Żukiewicz-Sobczak W. (2014). Dietary exposure to cadmium, lead and nickel among students from south-east Poland. Ann. Agric. Environ. Med..

[43] Dadar M., Adel M., Nasrollahzadeh S.H., Fakhri Y. (2017). Trace element concentration and its risk assessment in common fish (*Clupeonella cultriventris* caspia Bordin, 1904) from southern basin of Caspian Sea. Toxin. Rev..

[44] Barone G., Storelli A., Garofalo R., Busco V.P., Quaglia N.C., Centrone G., Storelli M.M. (2015). Assessment of mercury and cadmium via seafood consumption in Italy: Estimated dietary intake (EWI) and target hazard quotient (THQ). Food Addit. Contam. Part A.

[45] Ruiz-Guzmán J.A., Marrugo-Negrete J.L., Díez S. (2014). Human exposure to mercury through fish consumption: Risk assessment of riverside inhabitants of the Urrá Reservoir, Colombia. Hum. Ecol. Risk Assess..

[46] Jędruch A., Bełdowska M., Ziółkowska M. (2019). The role of benthic macrofauna in the trophic transfer of mercury in a low-diversity temperate coastal ecosystem (Puck Lagoon, southern Baltic Sea). Environ. Monit. Assess..

[47] Djedjibegovic J., Marjanovic A., Tahirovic D., Caklovica K., Turalic A., Lugusic A., Omeragic E., Sober M., Caklovica F. (2020). Heavy metals in commercial fish and seafood products and risk assessment in adult population in Bosnia and Herzegovina. Sci. Rep..

